# Feasibility of a Comprehensive Home Monitoring Program for Sarcoidosis

**DOI:** 10.3390/jpm9020023

**Published:** 2019-05-05

**Authors:** Catharina C. Moor, Yasmin Gür-Demirel, Marlies S. Wijsenbeek

**Affiliations:** Department of Respiratory Medicine, Erasmus Medical Center Rotterdam, 3015 GD Rotterdam, The Netherlands; c.moor@erasmusmc.nl (C.C.M.); y.gur-demirel@erasmusmc.nl (Y.G.-D.)

**Keywords:** lung, sarcoidosis, eHealth, home monitoring, wearable devices, feasibility, patient experiences

## Abstract

Sarcoidosis is a chronic, heterogeneous disease which most commonly affects the lungs. Currently, evidence-based and individually tailored treatment options in sarcoidosis are lacking. We aimed to evaluate patient experiences with a home monitoring program for sarcoidosis and assess whether home monitoring is a feasible tool to enhance personalized treatment. Outpatients with pulmonary sarcoidosis tested the home monitoring program “Sarconline” for one month. This is a secured personal platform which consists of online patient-reported outcomes, real-time wireless home spirometry, an activity tracker, an information library, and an eContact option. Patients wore an activity tracker, performed daily home spirometry, and completed patient-reported outcomes at baseline and after one month. Patient experiences were evaluated during a phone interview. Ten patients were included in the study. Experiences with the home monitoring program were positive; 90% of patients considered the application easy to use, none of the patients found daily measurements burdensome, and all patients wished to continue the home monitoring program after the study. Mean adherence to daily spirometry and activity tracking was, respectively, 94.6% and 91.3%. In conclusion, a comprehensive home monitoring program for sarcoidosis is feasible and can be used in future research and clinical practice.

## 1. Introduction

Sarcoidosis is a chronic, granulomatous disorder of unknown etiology with a heterogeneous presentation and disease course. This multisystem disease can be localized in almost any organ but most commonly affects the lungs and lymphatic system [1]. Symptoms such as dyspnea, persistent cough, fatigue, and physical limitations negatively affect the quality of life (QoL) of patients and often lead to stress and even anxiety, depression, and social isolation [2,3,4]. Oral corticosteroids are the mainstay of treatment for patients with significant symptoms or impaired organ function. However, there is a lack of evidence-based treatment regimens for sarcoidosis, and little information is available regarding long-term effects, optimal duration, and dosage of medication [1,5]. In practice, this may result in under- or overtreatment, with often unnecessary side-effects. In approximately 70% of patients, sarcoidosis resolves spontaneously or after treatment; in about one-third of patients, sarcoidosis becomes chronic and progressive [1]. 

Recently, much research effort has been put into new “omic” techniques and the identification of biomarkers to predict disease progression in sarcoidosis to determine who is likely to have spontaneous resolution, who should be treated, and who will respond to therapy. However, there is still a long way to go before this could possibly be used to enable personalized treatment [6,7]. Personalized medicine should not only take these biological factors into account but also patient factors, such as preferences, lifestyle, comorbidities, and response to treatment [8,9]. New eHealth technologies could play an important role in facilitating personalized care by frequent monitoring of lung function, activity, symptoms, side-effects, and QoL at home at a low burden for patients [10,11,12]. In that way, not only are more insights gained in the disease course, but therapies can also be better tailored. In an interactive survey on needs and preferences in sarcoidosis, the majority of patients supported the idea of managing their personal health-related data online. Almost all patients reported that they would be willing to measure lung function at home to enhance personalized treatment [13]. 

**Figure 1:**
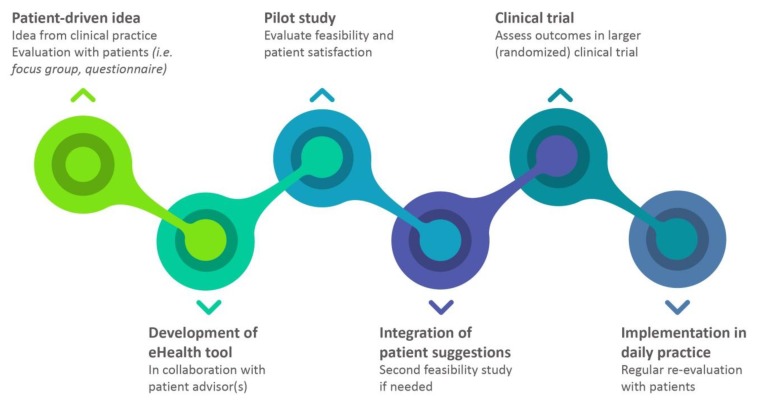
Framework for development of eHealth tools in close collaboration with patients. The current project falls within the pilot study phase.

An observational study showed that home spirometry was feasible and allowed for early detection of steroid treatment effects in sarcoidosis [14]. However, patients recorded their lung function and symptoms in a paper diary, which made it impossible to respond directly to changes in lung function or symptoms. Recently, we have developed a home monitoring program together with idiopathic pulmonary fibrosis (IPF) patients, including real-time wireless home spirometry and online recording of symptoms and side-effects [15]. Development of eHealth tools in close collaboration with patients may result in better outcomes, because the final “product” will be customized to patients' needs and wishes [11,16]. We have adapted this home monitoring program for sarcoidosis. In the current study, we aimed to evaluate patient satisfaction and the feasibility of this home monitoring program, and to assess its possible role for future clinical trials and daily practice (Figure 1).

## 2. Materials and Methods

### 2.1. Study Design and Population

This was a prospective observational study at the pulmonary department of the Erasmus Medical Center, Rotterdam, the Netherlands, a tertiary referral center for sarcoidosis. Consecutive outpatients with sarcoidosis were recruited prospectively at the outpatient clinic. Inclusion criteria were a diagnosis of sarcoidosis according to the ATS/ERS/WASOG criteria with pulmonary involvement and age above 18 years [1]. Patients were excluded if they were not able to speak, write, and read in Dutch, had no internet access at home, or no compatible smartphone/tablet. This study was approved by the local ethics committee (MEC-2018-1536). All patients gave online informed consent. 

### 2.2. Study Procedures

Patients were invited to test the home monitoring program “Sarconline” (www.sarconline.nl) for one month. The test period consisted of daily home spirometry, activity tracking, and recording of symptoms and patient-reported outcomes (PROMs) at baseline and after one month. After one month, the test period was evaluated with patients during a phone interview. 

#### 2.2.1. Home Monitoring Program

**Figure 2:**
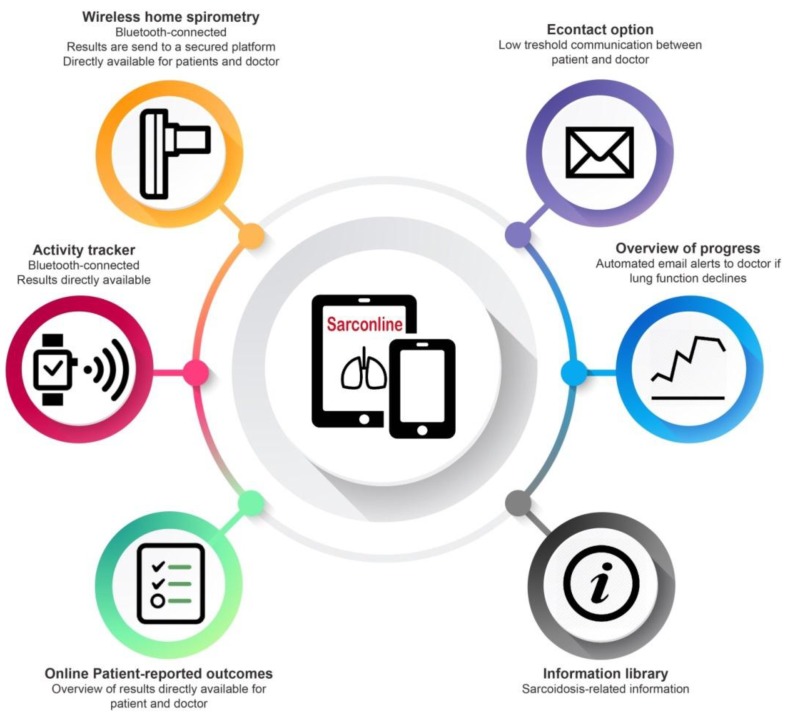
Overview of home monitoring program “Sarconline” with different components.

Sarconline is an online eHealth application developed for patients with sarcoidosis (Curavista, The Netherlands). It consists of a secured personal platform, in which patients can keep track of their own health-related data, such as pulmonary function tests, activity, quality of life questionnaires, symptoms, and medication (Figure 2). Patients directly see a graphical overview of their data. There is a possibility to communicate with the healthcare team by using the email functionality (eContact). Sarconline also contains news and information about sarcoidosis and links to useful websites. The patient owns the data and determines which healthcare providers can access his or her data. Data are stored on a secured and approved datacenter with ISO27001 certification, following European safety legislations. 

#### 2.2.2. Home Spirometry

Patients received a Bluetooth-enabled handheld spirometer (MIR, Spirobank Smart, Italy) to measure pulmonary function at home. This spirometer measures forced vital capacity (FVC), forced expiratory volume in 1 s (FEV1), and peak expiratory flow (PEF). Patients were requested to send their daily results directly to Sarconline via a secured encrypted connection using a five-digit personal code. If FVC declines for three consecutive days (relative decline ≥10%), an automated email alert is sent to the research team. At baseline, patients were trained on how to use the home monitoring program, including the handheld spirometer, for approximately 20 min. Patients were considered adequately trained if they could perform three measurements with less than a 150-mL difference in the two best FVCs and difference with hospital-based spirometry was less than 10%. 

#### 2.2.3. Activity Tracking

Steps per day, activity level, and calories were measured with a Bluetooth-enabled wrist-worn activity tracker (Fitbit Flex 2, FitBit, Inc., San Francisco, CA, USA). Patients were instructed to wear the activity tracker at daytime. Patients who wished to track their sleep could also wear the activity tracker at night time. The Fitbit activity tracker has incorporated behavior change techniques for activity and sleep, intended to stimulate long-term behavior change (i.e., goal-setting, alerts, and rewards) [17]. Activity tracker data were imported in Sarconline. 

#### 2.2.4. Patient-Reported Outcome Measures (PROMs)

Patients were asked to complete a number of QoL-related questionnaires and symptom scores at baseline and after one month. The King's Sarcoidosis Questionnaire (KSQ) assesses health status in patients with sarcoidosis. It comprises 29 items in 5 subdomains: general health status, lung, medication, skin, and eyes [18]. The Euroqol-5D-5L (EQ5D-5L) comprises five questions on the domains of mobility, self-care, daily activities, pain, and mood, and a Visual Analogue Scale on general health-status [19]. The Hospital Anxiety and Depression Scale (HADS) comprises a seven-item depression scale and a seven-item anxiety scale. The scores range from 0 to 21 for either anxiety or depression. The cut-off point of 8/21 is identified for either anxiety or depression [20]. The fatigue assessment scale (FAS) is a 10-item self-administered questionnaire about fatigue in patients with sarcoidosis. The score ranges from 5 to 50 points, with a score of ≥22 points as the cut-off for fatigue [21]. Patients were asked to complete weekly VASs on fatigue, dyspnea, cough, and general wellbeing, with scores ranging from 0 to 10. 

#### 2.2.5. Phone Interview

During a phone interview, patients were questioned about their opinion towards the home monitoring program. Satisfaction, feasibility of the program, and ease of use of the application and different devices were evaluated with patients. Furthermore, patients were asked whether they encountered (technical) problems, wished to continue home monitoring after the pilot, and had any suggestions or advice to improve the system. 

### 2.3. Statistical Analysis

Data are presented as median (range) or mean (SD). Adherence to home spirometry and activity tracking was assessed by dividing the total number of measurements by the total number of days and is expressed in percentage (%). Correlations between lung function (hospital and home), activity level, and symptoms were analyzed with Pearson's correlation coefficients. Differences between results of patient-reported outcomes at baseline and after one month were evaluated with the Wilcoxon signed rank test. Patient experiences, satisfaction, and use of the home monitoring program is qualitatively described. Statistical analyses were performed with SPSS version 24. A *p*-value < 0.05 was considered statistically significant. Because this was a pilot study, no formal power calculation could be performed. We aimed to include 10 patients based on a previous pilot study in IPF [15].

## 3. Results

Of 11 consecutive outpatients invited to participate, 10 patients with a broad range in age, time since diagnosis, and disease severity were enrolled in this study. One patient was excluded because she did not bring a compatible smartphone. Baseline characteristics of patients are described in Table 1. 

### 3.1. Home-Based Assessments

All patients managed to complete online PROMs, perform daily home spirometry, and track their activity at home. Mean adherence to daily spirometry was 94.6% (SD: 9). Home spirometry measurements highly correlated with in-hospital measurements of FVC (*r* = 0.97, *p* < 0.001) and FEV1 (*r* = 0.96, *p* < 0.001). One subject with severe airflow obstruction (FEV1/FVC of 27%) had consistently much lower home FVC results compared with hospital FVC (difference of 0.65 L or 18%). When leaving this subject out, median difference between hospital and home spirometry was 0.26 L (0.08–0.55 L), with overall lower readings for home spirometry in 78% of patients. Within-subject reproducibility was assessed; median SD of 28 FVC measurements was 0.17 L (0.09–0.38 L) and the median coefficient of variation was 5.78% (2%–8%).

Results of PROMs, lung function, and activity are summarized in Table 2. In one patient, daily activity could not be analyzed because there were technical difficulties with sending the Fitbit results to the secured platform. For the other patients, mean adherence to daily activity tracking was 91.3% (SD: 19); seven patients had 100% adherence. One patient measured activity on only 43% of days because he was not allowed to wear the wrist-worn tracker at work.

Symptoms measured by HADS, FAS, and VAS and QoL measured by KSQ and EQ5D-5L were not significantly different at baseline compared to month one. Moreover, no changes in daily step count and home-based FVC were observed during the study period. There was no correlation between lung function and mean daily step count for FVC (*r* = −0.38, *p* = 0.31) and a trend toward significance for diffusion capacity of the lung for carbon monoxide (DLCO) (*r* = 0.66, *p* = 0.08). Furthermore, no correlations were found between activity level and PROM scores as well as lung function and PROM scores. 

### 3.2. Patient Experiences

**Figure 3:**
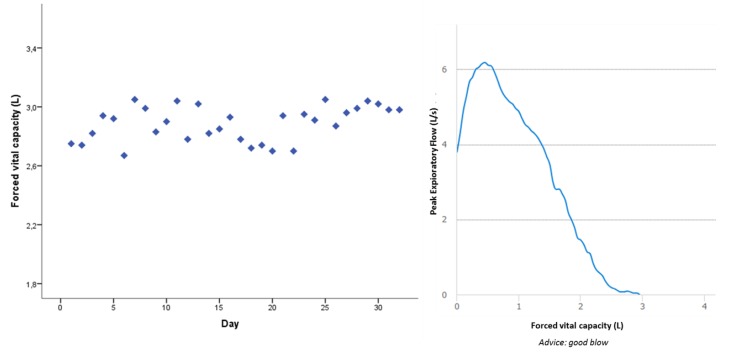
Measurements of FVC of an individual patient during one month, including one example of a flow volume loop.

Overall, patient experiences of the home monitoring program were positive. Almost all patients (90%) considered the application easy to use. None of the patients considered daily spirometry, activity tracking, and reporting of PROMs burdensome. All patients wished to continue the use of the home monitoring program after the test period. The vast majority of patients (90%) answered that they would be willing to measure daily lung function for a prolonged period of time to enhance individually tailored treatment, to evaluate response to therapy, or for study purposes. One patient mentioned that it could possibly be distressing to be confronted with your disease every day; this patient would prefer home spirometry at a weekly interval. Patients responded that it was very useful for them to see a daily overview of their lung function; this gave better insights into the effects of medication and the progression of their disease (Figure 3). The direct feedback on the quality of the measurement was perceived as useful guidance.

All patients endorsed the usefulness of activity tracking, as this stimulated them to be more active. Two patients mentioned that their activity level corresponded better with their overall functioning than lung function alone. All patients used the Fitbit to track sleep; 70% of patients found that this provided good insights in their fatigue and sleep patterns.

At baseline, patients were asked to fill in their personal goal for the upcoming period and their plan to reach this goal. Five patients wished to improve their dyspnea, four patients fatigue, and one patient general wellbeing. A few examples of how patients planned to reach these goals were to sport once a week, live a more regular life, and good adherence to medication. In the home monitoring program, patients could track their personal goal over time. One patient mentioned that this was an extra incentive to change her behavior. Most patients believed that establishing a personal goal can be of added value, especially when treatment is changed or when symptoms get worse.

During the test period, 50% of patients used the eContact option; 16 eConsultations were sent in total. Patients appreciated the short lines of communication: “If I have a problem and send a message, I get a really quick response”. Patients provided various suggestions for improvement and feedback on what they felt was missing in the home monitoring program. More disease-specific information and information about lung function was desired by three patients. Some patients would like more wearable devices integrated in the app. Four patients mentioned that it could be interesting to measure oxygen saturation at home, and two patients would like to monitor their heartrate more closely. Some minor technical problems occurred during the test period, such as problems with sending activity results via the app, difficulties with completing a questionnaire, and a bad Bluetooth connection between spirometer and app. A selection of more detailed patient quotes from the evaluation interviews is given in Table 3. 

## 4. Discussion

To our knowledge, this is the first study evaluating feasibility and patient experiences with a comprehensive online home monitoring program for sarcoidosis. Patient satisfaction and adherence to daily spirometry and activity tracking were high. All patients wished to continue the use of the home monitoring program after the study. Only a small number of technical problems occurred, and patients had useful suggestions for improving the system. These suggestions, such as the possibility to report side-effects, explanation about lung function testing, and adjustable reminders will be implemented in the program. The high patient satisfaction and compliance with home-based assessments is in line with pilot studies on home monitoring in other chronic diseases [15,22,23,24,25]. Previous studies also showed that evaluation with patients yields valuable insights into how to enhance personalized medicine through eHealth solutions, with key elements described in Table 4 [11,15,16,22,23,24].

Other studies using home spirometry in sarcoidosis and pulmonary fibrosis showed comparable results regarding correlation with hospital FVC, reproducibility, and overall lower results for home spirometry [14,15,26]. The current study showed a slightly higher variability between daily FVC measurements, possibly because patients measured their lung function at different times during the day. Besides, the patients who used inhaled bronchodilators did not always perform spirometry consequently before or after their medication. Hence, patients should be instructed to perform home spirometry at the same time every day, also taking into account medication use. Whether home measurements of FVC are reliable in patients with severe airflow obstruction and low exhaled flow should be studied further. It could be speculated that equipment-related factors can play a role in these patients, as the turbine flow sensors of home spirometers are probably less capable of detecting very low flow rates. Patients considered home monitoring easy and not burdensome at all, which are promising results for future applications. Real-time home spirometry in sarcoidosis can potentially be used in upcoming clinical trials evaluating the efficacy of (new) sarcoidosis treatments. If future and larger studies also show positive experiences with the use of a home spirometer, this could pave the way for use in clinical care. Home spirometry could be an attractive method to evaluate pulmonary improvement after starting or switching treatment, allowing for early tapering of medication in individual patients and facilitating timely detection of disease deterioration. 

A few observational studies evaluated activity levels in patients with sarcoidosis using an activity tracker for a short period of time (5–7 days) [27,28,29]. The current study shows that sarcoidosis patients find it feasible and not burdensome to collect activity data for a sustained period. The range in daily step count in previous studies in sarcoidosis was between 4566 and 7490 steps. The daily step count in our study was somewhat higher; patients walked on average 9780 steps per day over a one-month period. This could be partly due to differences in sarcoidosis severity, as one previous study only included patients with chronic stage IV sarcoidosis [28]. Moreover, patients in our study mentioned that they were more motivated to walk because of the activity tracker. In contrast to previous studies in sarcoidosis, the activity tracker used in the current study is connected to a mobile application, with integrated reminders and alerts to stimulate activity. A recent survey amongst users of activity trackers showed that wearing an activity tracker increases activity levels for a prolonged period of time [30]. Studies concerning physical activity and rehabilitation in sarcoidosis are scarce; nonetheless, current evidence suggests that increased physical activity leads to better health outcomes [31]. Thus, activity trackers with incorporated behavior change techniques could potentially be of added value in future interventional studies in sarcoidosis to enhance physical activity as part of a comprehensive home monitoring or rehabilitation program. For this purpose, integration of other data, such as heart rate measurements, should also be studied in sarcoidosis patients.

The main limitation of this study is the inclusion of a limited number of patients from one tertiary referral center. Nevertheless, the number of participants was sufficient for evaluating feasibility and patient experiences with the home monitoring program. During the last evaluation interview, no new information emerged, meaning that data saturation was established. Moreover, a mixed group of patients with a broad range of age, disease severity, and treatment was included. The fact that all consecutive patients were willing to participate highlights the clinical applicability of home monitoring in sarcoidosis.

In conclusion, a comprehensive home monitoring program is feasible and can be used in sarcoidosis research. Home monitoring could also be attractive for use in daily care, though studies are needed to evaluate its role and additive value. Potentially, home monitoring may enable timely recognition and response to changes in symptoms, lung function, and activity. Especially in a heterogeneous disease such as sarcoidosis, home monitoring may pave the way for better individually tailored treatment, enhanced self-management, and improved quality of life. 

## Figures and Tables

**Table 1:** Baseline characteristics of study patients (*n* = 10).

Age	53 (31–68)
Women	4 (40)
Ethnicity	
Caucasian	9 (90)
Surinamese Hindi	1 (10)
Time since diagnosis, year	5 (0–15)
Multiorgan involvement	6 (60)
BMI, kg/m^2^	27 (19–35)
Medication	
Prednisone	9 (90)
Methotrexate	6 (60)
Other	3 (30)
Pulmonary function	
FVC % predicted	86 (69–105)
FVC (L)	3.50 (2.53–6.47)
FEV1 % predicted	81 (25–97)
FEV1 (L)	2.60 (0.96–3.68)
FEV1/FVC (%)	72 (27–89)
DLCO (%)	74 (44–96)

Data are presented as median (range) or *n* (%). BMI = body mass index, FVC = forced vital capacity, FEV1 = forced expiratory volume in 1 s, DLCO = diffusion capacity of the lung for carbon monoxide.

**Table 2:** Home-based assessment of study patients (*n* = 10).

Daily step count	9781 (4355–17274)
Active, min/day	309 (146–484)
Light activity, min/day	263 (124–401)
Home-based FVC (L)	3.3 (2.4–6.2)
Home-based FEV1 (L)	2.5 (0.8–3.4)
EQ5D-5L index value	0.81 (0.1–0.92)
EQ5D-5L VAS	76 (14–93)
KSQ General health	62 (36–77)
KSQ Lung	61 (37–72)
HADS anxiety	7 (2–12)
HADS depression	6 (1–11)
FAS	25 (17–37)

Data are presented as median (range). Activity and lung function data are mean results for one month; results of patient-reported outcomes are measured at baseline. EQ5D-5L = Euroqol-5D-5L, VAS = visual analogue scale, KSQ = King's sarcoidosis questionnaire, HADS = hospital anxiety and depression scale, FAS = fatigue assessment scale.

**Table 3:** Selection of patient quotes from the evaluation interview.

“It is very difficult to tell exactly how you felt four weeks ago. By completing the questionnaires, my personal goal, and symptoms on a regular basis, I get a better overview of my disease over time.”
“I think this app gives much more details about my health than the regular outpatient clinic visit every three months. This information could also be very helpful for my doctor and nurses.”
“For me, the app contains enough information and devices at the moment. Otherwise, I might become too much focused on my disease.”
“I use inhaled medication; this had a direct positive effect on my lung function. I appreciated it very much that I was able see this directly at home. Now, I really know why I have to use it.”
“I have quite some side-effects from my medication. I would appreciate if I also could monitor my side-effects in the app.”
“It is a reassuring idea that the healthcare team monitors you at a distance, and that they directly see it if your lung function declines.”
“Reminders on my email to perform my measurements would be very helpful for me; otherwise, I forget it sometimes. But I understand that other patients probably won't need those reminders.”
“When I did not reach my step goal at the end of the day, I went outside to walk around some more. Seeing my activity was very motivating.”
“Sometimes, it was frustrating to see my step count, especially on the days that I was very tired and not feeling well. I wished I could walk more, but on some days, that was just not possible.”
“Everything together, the questionnaires, overview of symptoms, lung function, and activity gave a good total picture of my health.”
“It would be helpful to receive some more information or education about lung function. What do the different tests measure exactly and how should I interpret the results? Maybe it is an idea to make an information movie about this. For me, that is easier to understand than text.”
“My sarcoidosis is very stable at the moment. I think home monitoring would be more useful if your disease is getting worse, or if you start with new medication.”
“I have had some technical difficulties with the connection between spirometer and the app, but when I called the helpdesk, they could help me out.”
“If possible, I would also like to track my heart rate. If I don’t feel good, my heart rate goes up very fast. I think this would give extra information about my physical condition.”

**Table 4:** Key factors for integrating personalized care in eHealth.

Application customized to patients’ needs and wishes
Low threshold communication (i.e., eContact or video contact)
Patient education
Real-time availability of data for patients and healthcare providers
Adjustable email reminders for patients and healthcare providers
Integration of personal goal
Low burden for patients and healthcare providers

## References

[1] Costabel U., Hunninghake G.W. (1999). Ats/ers/wasog statement on sarcoidosis. Sarcoidosis statement committee. American thoracic society. European respiratory society. World association for sarcoidosis and other granulomatous disorders. Eur. Respir. J..

[2] Goracci A., Fagiolini A., Martinucci M., Calossi S., Rossi S., Santomauro T., Mazzi A., Penza F., Fossi A., Bargagli E. (2008). Quality of life, anxiety and depression in sarcoidosis. Gen. Hosp. Psychiatry.

[3] Judson M.A. (2017). Quality of life in sarcoidosis. Semin. Respir. Crit. Care Med..

[4] Drent M., Strookappe B., Hoitsma E., De Vries J. (2015). Consequences of sarcoidosis. Clin. Chest Med..

[5] Paramothayan N.S., Lasserson T.J., Jones P.W. (2005). Corticosteroids for pulmonary sarcoidosis. Cochrane Database Syst. Rev..

[6] Landi C., Carleo A., Cillis G., Rottoli P. (2018). Sarcoidosis: Proteomics and new perspectives for improving personalized medicine. Expert Rev. Proteom..

[7] Spagnolo P., Oldham J.M., Jones M.G., Lee J.S. (2017). Personalized medicine in interstitial lung diseases. Curr. Opin. Pulm. Med..

[8] Ziegelstein R.C. (2015). Personomics. JAMA Intern. Med..

[9] Moor C.C., Heukels P., Kool M., Wijsenbeek M.S. (2017). Integrating patient perspectives into personalized medicine in idiopathic pulmonary fibrosis. Front. Med. (Lausanne).

[10] Wicks P., Stamford J., Grootenhuis M.A., Haverman L., Ahmed S. (2014). Innovations in e-health. Qual. Life Res..

[11] Velardo C., Shah S.A., Gibson O., Clifford G., Heneghan C., Rutter H., Farmer A., Tarassenko L., Team E.C. (2017). Digital health system for personalised copd long-term management. BMC Med. Inform. Decis. Mak..

[12] Steven Kohn M. (2018). Editorial commentary: Wearable devices and personalized healthcare. Trends Cardiovasc. Med..

[13] Moor C.C., van Manen M.J.G., van Hagen P.M., Miedema J.R., van den Toorn L.M., Gur-Demirel Y., Berendse A.P.C., van Laar J.A.M., Wijsenbeek M.S. (2018). Needs, perceptions and education in sarcoidosis: A live interactive survey of patients and partners. Lung.

[14] Broos C.E., Wapenaar M., Looman C.W.N., In 't Veen J., van den Toorn L.M., Overbeek M.J., Grootenboers M., Heller R., Mostard R.L., Poell L.H.C. (2018). Daily home spirometry to detect early steroid treatment effects in newly treated pulmonary sarcoidosis. Eur. Respir. J..

[15] Moor C.C., Wapenaar M., Miedema J.R., Geelhoed J.J.M., Chandoesing P.P., Wijsenbeek M.S. (2018). A home monitoring program including real-time wireless home spirometry in idiopathic pulmonary fibrosis: A pilot study on experiences and barriers. Respir. Res..

[16] Simpson A.J., Honkoop P.J., Kennington E., Snoeck-Stroband J.B., Smith I., East J., Coleman C., Caress A., Chung K.F., Sont J.K. (2017). Perspectives of patients and healthcare professionals on mhealth for asthma self-management. Eur. Respir. J..

[17] Duncan M., Murawski B., Short C.E., Rebar A.L., Schoeppe S., Alley S., Vandelanotte C., Kirwan M. (2017). Activity trackers implement different behavior change techniques for activity, sleep, and sedentary behaviors. Interact. J. Med. Res..

[18] Patel A.S., Siegert R.J., Creamer D., Larkin G., Maher T.M., Renzoni E.A., Wells A.U., Higginson I.J., Birring S.S. (2013). The development and validation of the king's sarcoidosis questionnaire for the assessment of health status. Thorax.

[19] Brooks R. (1996). Euroqol: The current state of play. Health Policy.

[20] Zigmond A.S., Snaith R.P. (1983). The hospital anxiety and depression scale. Acta Psychiatr. Scand..

[21] De Vries J., Michielsen H., Van Heck G.L., Drent M. (2004). Measuring fatigue in sarcoidosis: The fatigue assessment scale (FAS). Br. J. Health Psychol..

[22] Schenkel F.A., Ganesh S., O'Conner J., Sadeghi R., Bembi M., Duong M., Barr M.L., Hackmann A.E. (2018). Pilot experience with a novel bluetooth tablet-based technology for home monitoring and education after lung transplantation. J. Heart Lung Transpl..

[23] de Jong M., van der Meulen-de Jong A., Romberg-Camps M., Degens J., Becx M., Markus T., Tomlow H., Cilissen M., Ipenburg N., Verwey M. (2017). Development and feasibility study of a telemedicine tool for all patients with IBD: Myibdcoach. Inflamm. Bowel Dis..

[24] Odeh B., Kayyali R., Nabhani-Gebara S., Philip N., Robinson P., Wallace C.R. (2015). Evaluation of a telehealth service for COPD and HF patients: Clinical outcome and patients' perceptions. J. Telemed. Telecare.

[25] Williams V., Price J., Hardinge M., Tarassenko L., Farmer A. (2014). Using a mobile health application to support self-management in COPD: A qualitative study. Br. J. Gen. Pract..

[26] Russell A.M., Adamali H., Molyneaux P.L., Lukey P.T., Marshall R.P., Renzoni E.A., Wells A.U., Maher T.M. (2016). Daily home spirometry: An effective tool for detecting progression in idiopathic pulmonary fibrosis. Am. J. Respir. Crit. Care Med..

[27] Bahmer T., Watz H., Develaska M., Waschki B., Rabe K.F., Magnussen H., Kirsten D., Kirsten A.M. (2018). Physical activity and fatigue in patients with sarcoidosis. Respiration.

[28] Froidure S., Kyheng M., Grosbois J.M., Lhuissier F., Stelianides S., Wemeau L., Wallaert B. (2019). Daily life physical activity in patients with chronic stage IV sarcoidosis: A multicenter cohort study. Health Sci. Rep..

[29] Pilzak K., Zebrowska A., Sikora M., Hall B., Lakomy O., Kostorz S., Ziora D., Jastrzebski D. (2018). Physical functioning and symptoms of chronic fatigue in sarcoidosis patients. Adv. Exp. Med. Biol..

[30] Maher C., Ryan J., Ambrosi C., Edney S. (2017). Users' experiences of wearable activity trackers: A cross-sectional study. BMC Public Health.

[31] Strookappe B., Saketkoo L.A., Elfferich M., Holland A., De Vries J., Knevel T., Drent M. (2016). Physical activity and training in sarcoidosis: Review and experience-based recommendations. Expert Rev. Respir. Med..

